# Sequence-Specific Targeting of Bacterial Resistance Genes Increases Antibiotic Efficacy

**DOI:** 10.1371/journal.pbio.1002552

**Published:** 2016-09-15

**Authors:** Dilay Hazal Ayhan, Yusuf Talha Tamer, Mohammed Akbar, Stacey M. Bailey, Michael Wong, Seth M. Daly, David E. Greenberg, Erdal Toprak

**Affiliations:** 1 Green Center for Systems Biology, University of Texas Southwestern Medical Center, Dallas, Texas, United States of America; 2 Department of Biochemistry and Molecular Biology, University of Massachusetts Amherst, Amherst, Massachusetts, United States of America; 3 Department of Molecular Biophysics, University of Texas Southwestern Medical Center, Dallas, Texas, United States of America; 4 Sarepta Therapeutics, Cambridge, Massachusetts, United States of America; 5 Harvard Medical School, Cambridge, Massachusetts, United States of America; 6 Department of Internal Medicine, University of Texas Southwestern Medical Center, Dallas, Texas, United States of America; 7 Department of Microbiology, University of Texas Southwestern Medical Center, Dallas, Texas, United States of America; 8 Department of Pharmacology, University of Texas Southwestern Medical Center, Dallas, Texas, United States of America; MIT, UNITED STATES

## Abstract

The lack of effective and well-tolerated therapies against antibiotic-resistant bacteria is a global public health problem leading to prolonged treatment and increased mortality. To improve the efficacy of existing antibiotic compounds, we introduce a new method for strategically inducing antibiotic hypersensitivity in pathogenic bacteria. Following the systematic verification that the AcrAB-TolC efflux system is one of the major determinants of the intrinsic antibiotic resistance levels in *Escherichia coli*, we have developed a short antisense oligomer designed to inhibit the expression of *acrA* and increase antibiotic susceptibility in *E*. *coli*. By employing this strategy, we can inhibit *E*. *coli* growth using 2- to 40-fold lower antibiotic doses, depending on the antibiotic compound utilized. The sensitizing effect of the antisense oligomer is highly specific to the targeted gene’s sequence, which is conserved in several bacterial genera, and the oligomer does not have any detectable toxicity against human cells. Finally, we demonstrate that antisense oligomers improve the efficacy of antibiotic combinations, allowing the combined use of even antagonistic antibiotic pairs that are typically not favored due to their reduced activities.

## Abstract 2

Reducing expression of the AcrAB-TolC efflux system of *Escherichia coli* by antisense-mediated translation inhibition increases the susceptibility of bacteria to a wide range of antibiotics.

## Author Summary

Antibiotic resistance is a global health threat. While genome sequencing and genetic manipulation tools have elucidated many resistance mechanisms, these tools have not yet been developed into successful therapeutics. One tool with such potential are peptide-conjugated phosphorodiamidate morpholino oligomers (PPMOs), which are synthetic DNA/RNA mimics that function as antisense mRNA translation inhibitors. In this paper we use PPMOs to increase antibiotic susceptibility of bacteria. First, we identify the AcrAB-TolC efflux system as a major intrinsic resistance mechanism in *E*. *coli*. Then by targeting the mRNA of each component of this efflux system with PPMOs, we identify an *acrA*-PPMO as the most effective antisense molecule. Treatment of bacteria with *acrA*-PPMO resulted in a 2- to 40-fold increase in antibiotic efficacy, prevented translation of the AcrA protein, and inhibited efflux of antibiotic molecules without being cytotoxic to human cells. Finally, we demonstrate that *acrA*-PPMO is efficacious in several pathogenic bacterial genera and enhances activity of both synergistic and antagonistic antibiotic pairs when used together. This work establishes that PPMOs can potentially be used to treat infections caused by antibiotic resistant bacteria.

## Introduction

Antibiotic resistance is an important public health problem that emerged shortly after the discovery of antibiotics [1,2]. Pathogenic bacteria are either intrinsically resistant to some antibiotics or they acquire resistance via spontaneous mutations or horizontal gene transfer. These resistance mechanisms include deactivation or modification of antibiotics, pumping out antibiotics via efflux pumps, protection of antibiotic targets, and mutations in the target enzymes that decrease antibiotic affinity [3]. Even though the majority of these resistance mechanisms are well characterized at the molecular level, there has been limited success at avoiding the evolution of resistance in the clinic. There is a growing need for entirely new tools and strategies in order to stop or slow the evolution of antibiotic resistance in clinical settings [4].

Recent advances in biology, particularly whole genome sequencing technologies and gene-editing tools, have enabled us to identify resistance-conferring genetic changes and perform genetic manipulations that can reverse evolved antibiotic resistance [5–7]. By using novel gene-editing tools such as CRISPR-CAS9 or engineered bacteriophages, it is now possible to edit bacterial genomes to modulate antibiotic sensitivity of bacteria and also design sequence-specific antimicrobials [8–10]. However, gene-editing tools are currently difficult to implement given the practical and ethical problems with mutating bacterial genomes within an infected human patient. Instead, we designed antisense oligomers, which target the mRNA of bacterial resistance genes, preventing translation in a sequence-specific manner [11].

Briefly, phosphorodiamidate morpholino oligomers (PMOs) are synthetic nucleotide oligomers made from six-membered morpholine rings joined together by phosphorodiamidate linkages. Each morpholine ring has a natural nucleobase attached (see [12] for PMO structure), and the oligomers are designed to bind complementary sequences in targeted mRNAs. PMOs are thought to exert their effects through translation inhibition as a result of steric hindrance when targeting bacterial mRNA within the close proximity of ribosome binding sequences [13]. Cell-penetrating peptides are conjugated to the phosphorodiamidate morpholino oligomers (PPMOs), which enhance uptake of the oligomer into the bacterial cell [14]. Unlike short RNA molecules, which are also being considered as therapeutic agents, the synthetic PMO backbone renders them resistant to being hydrolyzed by nucleases [12,15]. Previous reports have shown PPMOs to be bactericidal in vitro and in vivo in a number of gram-negative pathogens when targeting essential genes [11,16]. Here, we demonstrate that PPMOs inhibit several resistance-conferring genes, improving efficacy of several distinct antibiotic classes.

## Results

**Fig 1:**
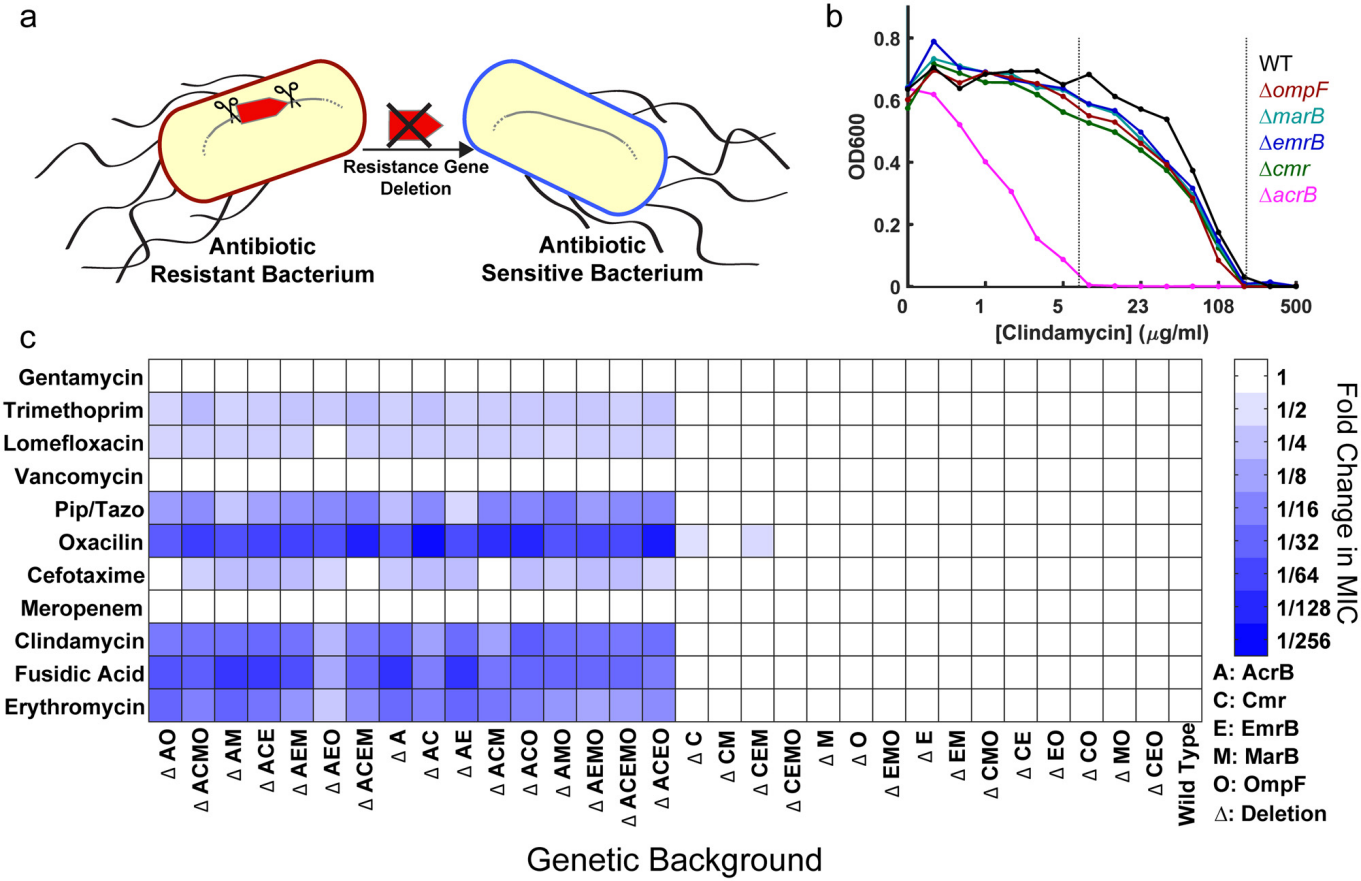
Systematic deletions of *E*. *coli* genes that encode for membrane proteins demonstrate that the AcrAB-TolC efflux system is the major machinery responsible for intrinsic antibiotic resistance. (A) Physical deletion of a resistance gene in a bacterium may render the bacterium antibiotic sensitive. (B) Representative MIC determination using final optical density at 600 nm (OD600) values at 22 h of incubation with the wild type (WT) *E*. *coli* and gene deletion mutants in increasing doses of clindamycin. The left vertical dashed line represents the MIC concentration for the *acrB* deletion mutant (magenta) while the right vertical dashed line represents the MIC for the remaining strains (WT and the *cmr*, *emrB*, *marB*, *ompF* deletion mutants). (C) Heat map showing the normalized mean MIC values for every strain, measured as in (B). MIC values were normalized using the wild type strain as the reference. All MIC measurements were run at least in duplicate and were found to be highly reproducible (S2B Fig). Relative change of the MIC (compared to WT) is depicted colorimetrically with blue representing statistically significant decreases (*p* < 0.05) in MIC and white representing nonsignificant changes in MIC. Intensity of the blue color indicates the magnitude of MIC change. MIC changes for only 11 of the 27 tested antibiotic compounds are shown here. The heat map for all antibiotics can be found in S2A Fig and the numerical MIC values can be found in S1 Table.

Active excretion of antibiotic molecules via efflux proteins or reducing the uptake of drug molecules by mutating or down-regulating membrane proteins (i.e., porins) are two of the common strategies that multidrug-resistant bacteria utilize in order to render antibiotics ineffective [3]. We, and others, have previously shown that several genes that encode membrane proteins in multidrug-resistant *E*. *coli* strains either accumulated point mutations or had changes in their regulation [5,17–20]. Thus, we hypothesized that deletion of such genes has the potential to increase antibiotic efficacy (Fig 1A). To test this idea, we selected five genes (*acrB*, *emrB*, *marB*, *ompF*, *cmr*) that encode membrane proteins in *E*. *coli* and deleted them with all 32 possible combinations in order to find the best target genes and quantify epistatic interactions between these gene deletions (S1 Fig) [3,21–24]. We then measured the minimum inhibitory concentrations (MICs) of these mutants against 27 different antibiotics (Fig 1B and 1C and S2 Fig). Deletion of *acrB*, alone or in combination with other genes, significantly increased the susceptibility of *E*. *coli* to several antibiotics, up to ~100-fold (Fig 1B and 1C and S2 Fig). However, deletions of the other genes (*emrB*, *marB*, *ompF*, *cmr*) did not significantly change antibiotic susceptibility (Fig 1B and 1C and S2 Fig). This suggests that these genes might be involved in acquired resistance when they are mutated or their regulation is altered, but they are not involved in intrinsic antibiotic resistance of *E*. *coli* against the 27 compounds we tested. Also, based on these measurements, there were no epistatic interactions between these gene deletions.

**Fig 2:**
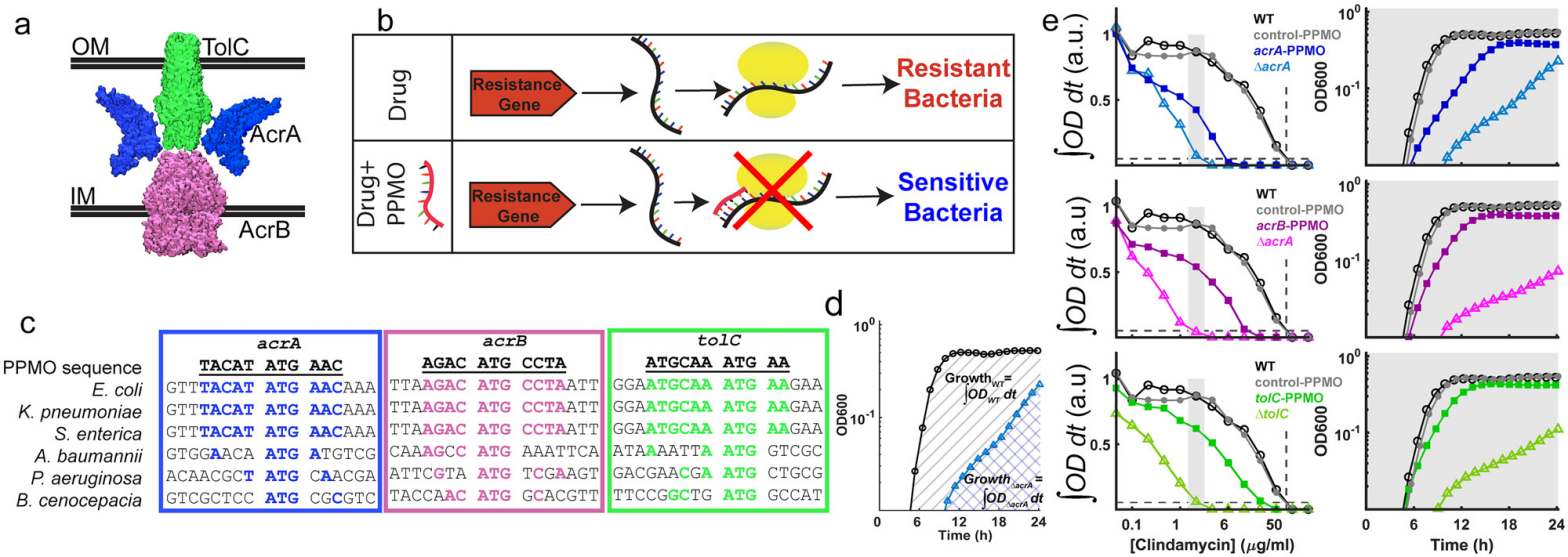
Targeting the genes that encode for the AcrAB-TolC efflux pump complex increases antibiotic susceptibility. (A) Cartoon representation of the AcrAB-TolC efflux system based on available crystal structures (PDB IDs: AcrA-2f1m, AcrB-2dhh, and TolC-1ek9). IM: Inner Membrane; OM: Outer Membrane. (B) PPMOs are antisense molecules that bind to complementary mRNAs and sterically interfere with their translation. Silencing resistance-conferring genes with this strategy leads to antibiotic susceptibility. (C) We have engineered three separate PPMOs in order to target the *acrA* (blue), *acrB* (magenta), and *tolC* (green) genes. These PPMOs target gene regions that span the start codons of the transcribed mRNA. Alignment of the *acrA*, *acrB*, and *tolC* genes of different bacterial genera demonstrate that the PPMO sequences, designed for *E*. *coli*, are also complementary in other pathogens. The overlapping nucleotides between the gene sequences and the PPMOs are highlighted in color. The PPMO sequences are homologous in *Klebsiella pneumoniae* and *Salmonella enterica* genes but have limited homology to the remaining bacterial species. (D) Growth of bacteria is quantified by calculating the area under the curve (AUC), which is simply integrating OD600 from 0 to 24 h (Materials and Methods). Area under the black (circles) and cyan lines (triangles) correspond to the growth of the wild type and *acrA* deletion *E*. *coli* strains, respectively, in a subinhibitory dose of clindamycin. (E) (Left) Dose response curves as a function of clindamycin concentration. Dose response curves are generated using the AUC values. Curves are labeled as untreated wild type *E*. *coli* (black lines, empty circles), with 10 μM control-PPMO (grey lines, filled circles), with 10 μM *acrA*-PPMO (top panel, blue lines, filled squares), *E*. *coli* with *acrA* deletion (top panel, cyan lines, empty triangles), with 10 μM *acrB*-PPMO (middle panel, magenta lines, filled squares), *E*. *coli* with *acrB* deletion (middle panel, pink lines, empty triangles), with 10 μM *tolC*-PPMO (bottom panel, dark green lines, filled squares), and *E*. *coli* with *tolC* deletion (bottom panel, light green lines, empty triangles). The horizontal dashed lines represent 95% growth inhibition, while the vertical lines represent the MIC value for WT. (Right) Sample OD600 versus time growth curves at the conditions shown within the grey shaded areas on the dose response curves (Left). Each line is interpolated, integrated and the AUC is normalized to the wild type growth in the absence of clindamycin. Dose response curves and corresponding MIC values for all 11 antibiotics may be found in S4 Fig and S2 Table, respectively.

The AcrAB-TolC efflux pump complex is among the best-characterized efflux pumps in *E*. *coli* and is composed of AcrB, the inner membrane antiporter, AcrA, the periplasmic adaptor protein, and TolC, the outer membrane channel (Fig 2A) [20,22,25–27]. Deleting *acrB* led to increased susceptibility (Fig 1B and 1C), so we deleted the two other genes (*acrA* and *tolC*) that together form the AcrAB-TolC efflux pump complex (Fig 2A) in *E*. *coli* to identify their contribution to the intrinsic antibiotic resistance of *E*. *coli*. Indeed, deletion of any of these three genes increased antibiotic sensitivity of *E*. *coli*, and loss of intrinsic antibiotic resistance due to gene deletions was reversed by plasmid complementation (S3 Fig).

We designed three PPMOs to target the *acrA*, *acrB*, and *tolC* genes, respectively (Fig 2A–2C). PPMOs were designed as 11-mers targeting gene regions near the translation start site with high-sequence specificity and low homology around other translation start sites in the *E*. *coli* genome (Fig 2C). We first tested the efficacy of these PPMOs by quantifying inhibitory effects of several antibiotic compounds against *E*. *coli* in the presence of PPMOs (Fig 2E, S4 Fig, S2 Table). All three PPMOs designed to target *acrA*, *acrB*, and *tolC* (hereafter called *acrA*-PPMO, *acrB*-PPMO, and *tolC*-PPMO) induced antibiotic sensitivity to multiple antibiotics, while a control PPMO (control-PPMO), which has a base sequence with low homology to *E*. *coli* translation start sites, had no effect on antibiotic sensitivity (Fig 2E and S4 Fig). Fig 2E demonstrates an example of this sensitizing effect with clindamycin, a protein synthesis inhibitor that is not commonly used against *E*. *coli* infections because of its high MIC. Enhancing the efficacy of clindamycin against *E*. *coli* is a significant finding that could make this drug potentially effective against gram-negative bacteria. Strikingly, use of *acrA*-PPMO (Fig 2E, blue line) showed a ~16-fold increase in clindamycin sensitivity comparable to the ~32-fold increase from deletion of the *acrA* gene (Fig 2E, cyan line). In almost every PPMO and antibiotic combination, the effects of *acrB*-PPMO and *tolC*-PPMO were less potent than the effect of *acrA*-PPMO (S4 Fig). Hence, we used *acrA*-PPMO for the rest of our experiments. The sensitization effect of *acrA*-PPMO varied between a 2- and 40-fold reduction in MIC, depending on the antibiotic compound (S4 Fig, S2 Table). It was surprising to find that, for certain antibiotic compounds, *acrA*-PPMO treatment did not adequately recapitulate the effect seen with the *acrA* deletion mutant (*e.g.*, compare chloramphenicol and oxacillin, S4 Fig).

**Fig 3:**
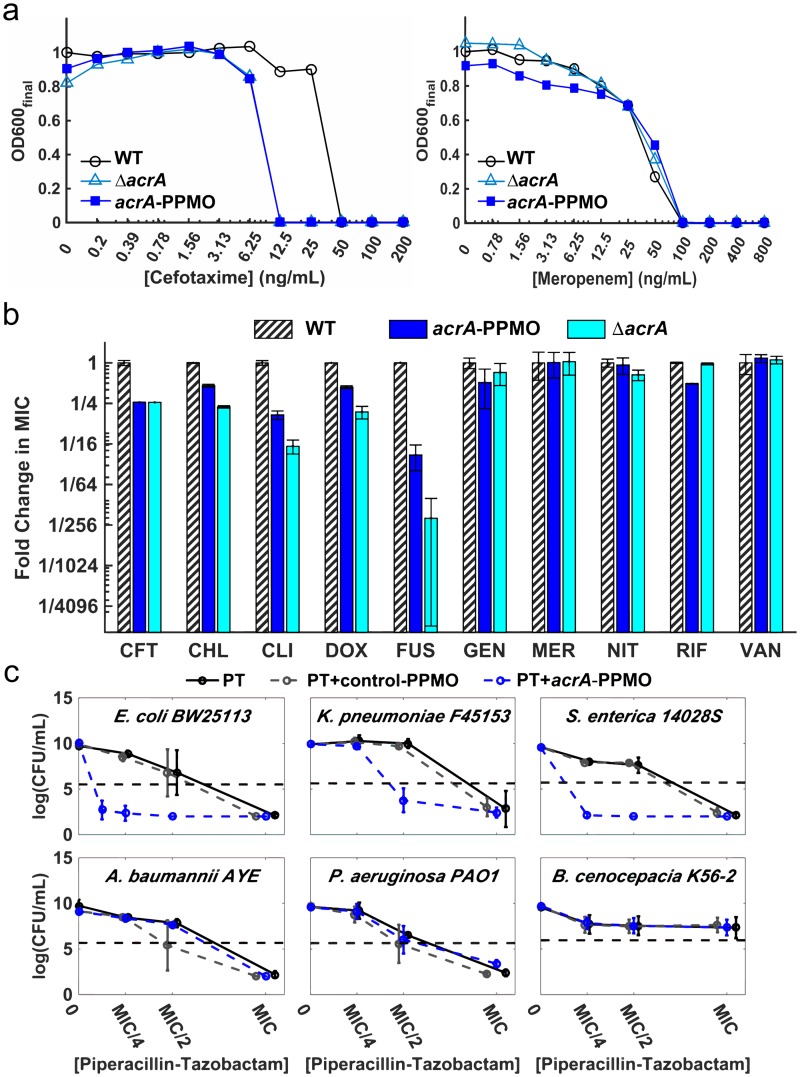
*acrA*-PPMO confers hypersensitivity to several antibiotics in a sequence-specific manner. (A) Sample antibiotic dose-response curves of *E*. *coli* in the absence of *acrA*-PPMO (black lines), in the presence of 10 μM *acrA*-PPMO (blue lines), and *E*. *coli* with *acrA* deletion (cyan lines). The MIC for each treatment is defined as the lowest concentration of antibiotic that results in a 95% reduction in the growth relative to the wild type *E*. *coli* in the presence of antibiotics (black lines). (B) Bar graphs of the measured fold changes in MIC values for wild-type *E*. *coli* in the absence of *acrA*-PPMO (black), 10 μM *acrA*-PPMO (blue), and with the *acrA* deletion (cyan). Abbreviations: CFT, cefotaxime; CHL, chloramphenicol; CLI, clindamycin; DOX, doxycycline; FUS, fusidic acid; GEN, gentamycin; MER, meropenem; NIT, nitrofurantoin; RIF, rifampicin; VAN, vancomycin. Every measurement was completed with four replicates, and error bars represent standard deviation. Phenotypic effects of *acrA* deletion and *acrA* silencing with *acrA*-PPMO are highly correlated (r = 0.94, *p* < 0.001, Pearson correlation test). (C) Killing of *E*. *coli* (BW25113), *K*. *pneumoniae* (F45153), *S*. *enterica* (14028S), *Acinetobacter baumannii* (AYE), *Pseudomonas aeruginosa* (PAO1), and *Burkholderia cenocepacia* (K56-2) by piperacillin-tazobactam alone (black line) or in combination with 10 μM control-PPMO (grey dashed line), or *acrA*-PPMO (blue dashed line) after 18 h incubation. The horizontal dashed line represents the inoculum (5 x 10^5^ CFU/mL) prior to incubation. The *x*-axis represents the normalized MIC concentration of piperacillin-tazobactam, corresponding to different MIC values for each pathogen. Error bars represent the standard deviations of the colony forming unit (CFU) counts obtained from at least four replicate measurements.

In order to further compare the phenotypic effects of the *acrA* deletion mutant to *acrA*-PPMO silencing, we tested the sensitization effect of *acrA*-PPMO with ten antibiotic compounds (Fig 3B). Five of these compounds were selected because they were more potent against *E*. *coli* strains with the *acrA* gene deletion, and the remaining five antibiotic compounds were selected since their efficacies were not expected to change based on the *acrA* gene deletion data (S2 Fig). Fig 3A presents two example antibiotic dose-response curves demonstrating the effect of *acrA*-PPMO when used together with cefotaxime or meropenem. Susceptibility of *E*. *coli* to cefotaxime increased when *acrA* was deleted or silenced (Fig 3A, left), whereas susceptibility to meropenem remained the same as the wild-type when *acrA* was deleted or silenced (Fig 3A, right). This pattern was consistent with the previous susceptibility data in all antibiotics tested (Fig 3B). In other words, the phenotypic effect of silencing the *acrA* gene with *acrA*-PPMO was indistinguishable from the phenotypic effect of *acrA* deletion (r = 0.94, *p* < 0.001, Pearson correlation test).

The nucleotide sequence near the translational start site is conserved between several bacterial genera for *acrA* (Fig 2C). We therefore hypothesized that *acrA*-PPMO would sensitize organisms with high sequence homology and would have no effect on organisms with low sequence homology. To demonstrate this, we compared the efficacy of *acrA*-PPMO against *E*. *coli*, *Klebsiella pneumoniae*, *Salmonella enterica*, *Acinetobacter baumannii*, *Pseudomonas aeruginosa*, and *Burkholderia cenocepacia*, which share between 36% and 100% *acrA* sequence homology to the *E*. *coli* target sequence (Fig 2C). In *E*. *coli*, time-kill assays after 18 h of exposure to subinhibitory concentrations (1/4 MIC) of piperacillin-tazobactam and 10 μM *acrA*-PPMO resulted in a three order of magnitude reduction in colony forming units (CFUs) from the starting inoculum compared to a three order of magnitude increase at the same concentration of antibiotic alone (Fig 3C). We demonstrated a similar sensitization effect of *acrA*-PPMO against *K*. *pneumoniae* and *S*. *enterica*, which share 100% sequence homology with *E*. *coli* (Fig 3C, top). Conversely, the *acrA*-PPMO had no activity against *A*. *baumannii*, *P*. *aeruginosa*, or *B*. *cenocepacia*, consistent with their lower (36%–45%) sequence homology (Fig 3C, bottom). Importantly, this demonstrated that the sensitization effect of *acrA*-PPMO was sequence-specific, and our strategy has the potential for being used against other pathogens.

**Fig 4:**
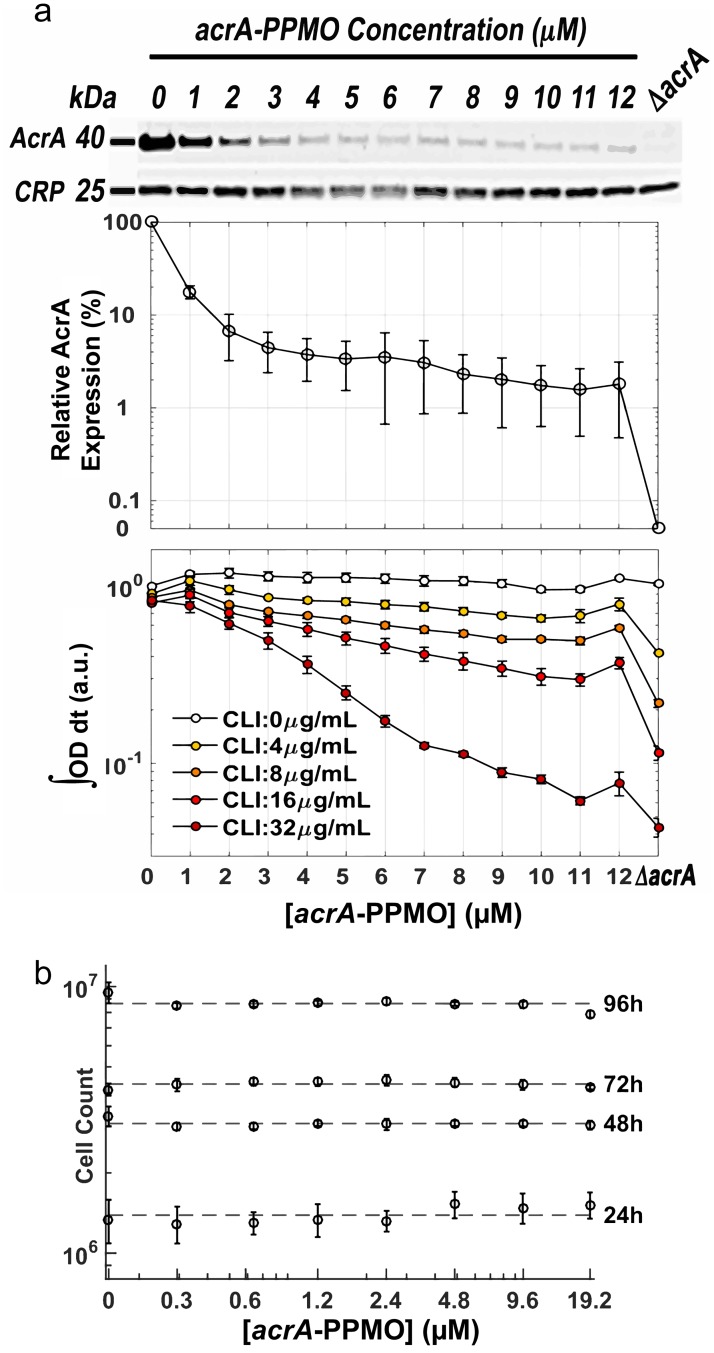
*acrA*-PPMO blocks *acrA* translation in a dose-dependent fashion and is nontoxic to HBEC3KT human cells. (A) AcrA expression in *E*. *coli* with increasing concentrations of *acrA*-PPMO was quantified using an anti-AcrA antibody (top panel). AcrA expression was normalized against the expression of cAMP receptor protein (CRP). Error bars represent the standard deviations of normalized AcrA protein levels for six experimental replicates (middle panel). *E*. *coli* growth in fixed concentrations of clindamycin with increasing concentrations of *acrA*-PPMO is calculated by calculating the AUC growth in different experimental conditions (bottom panel). Error bars represent the standard deviation of growth rate changes of four experimental replicates. (B) *acrA*-PPMO has nonsignificant levels of toxicity to HBEC3KT human cells. HBEC3KT human cells were incubated with increasing doses of *acrA*-PPMO, and the number of viable cells was determined (Cell-Titer-Glo, Promega) every 24 h for 4 d. Error bars represent the standard deviation of cell counts obtained from ten replicate experiments.

We quantified the AcrA protein expression in *E*. *coli* in increasing concentrations of *acrA*-PPMO (Fig 4A, top panel). AcrA protein levels decreased nearly 30-fold at *acrA*-PPMO concentrations greater than 3 μM (Fig 4A, middle panel). Control-PPMO had no effect on AcrA protein levels at 2 and 10 μM (S5 Fig). Residual expression of AcrA (~2% compared to untreated cells) is still detected even at the highest *acrA*-PPMO dose. We have also verified this effect by measuring growth rates of *E*. *coli* at different subinhibitory clindamycin concentrations using increasing concentrations of *acrA*-PPMO. Growth of *E*. *coli*, incubated with constant clindamycin concentrations, gradually decreased as *acrA*-PPMO concentration was increased (Fig 4A, bottom panel). This indicated that clindamycin susceptibility was correlated with the AcrA expression in *E*. *coli* (Fig 4A). Clindamycin sensitivity of *E*. *coli*, even at the highest concentrations of *acrA*-PPMO, was still lower than the sensitivity of the *E*. *coli* mutant with *acrA* deletion (Fig 4A, bottom panel), which is consistent with the residual AcrA expression even at the highest concentrations of *acrA*-PPMO (Fig 4A, top panel). To directly demonstrate that the *acrA*-PPMO’s inhibition of AcrA translation leads to reduced antibiotic efflux, we measured efflux of a DNA-binding dye, Hoechst 33342, which is also a substrate for the AcrAB-TolC complex [28]. The rate of fluorescence accumulation inside bacterial cytoplasm reflects the difference between concentration-dependent influx of Hoechst dye and the AcrAB-TolC-related efflux of the Hoechst dye. We found that *E*. *coli* cells treated with 2 and 10 μM of *acrA*-PPMO had significant increases in final fluorescence levels and fluorescence accumulation rates, comparable to the *acrA* deletion mutant (S6 Fig). This surrogate measure suggests that the efflux of antibiotic compounds is qualitatively similar to the efflux of the Hoechst dye; however, the magnitude of efflux will be specific to the chemical structure of particular antibiotics. Finally, we tested the toxicity of *acrA*-PPMO against human lung epithelial cells using a cell viability assay (Fig 4B). Even at 19.2 μM, *acrA*-PPMO had no significant toxicity at the end of 4 d. These observations provide clear evidence that *acrA*-PPMO is a promising agent that works as an efficient antibiotic adjuvant by preventing AcrA translation and therefore preventing efflux in a sequence-specific way.

**Fig 5:**
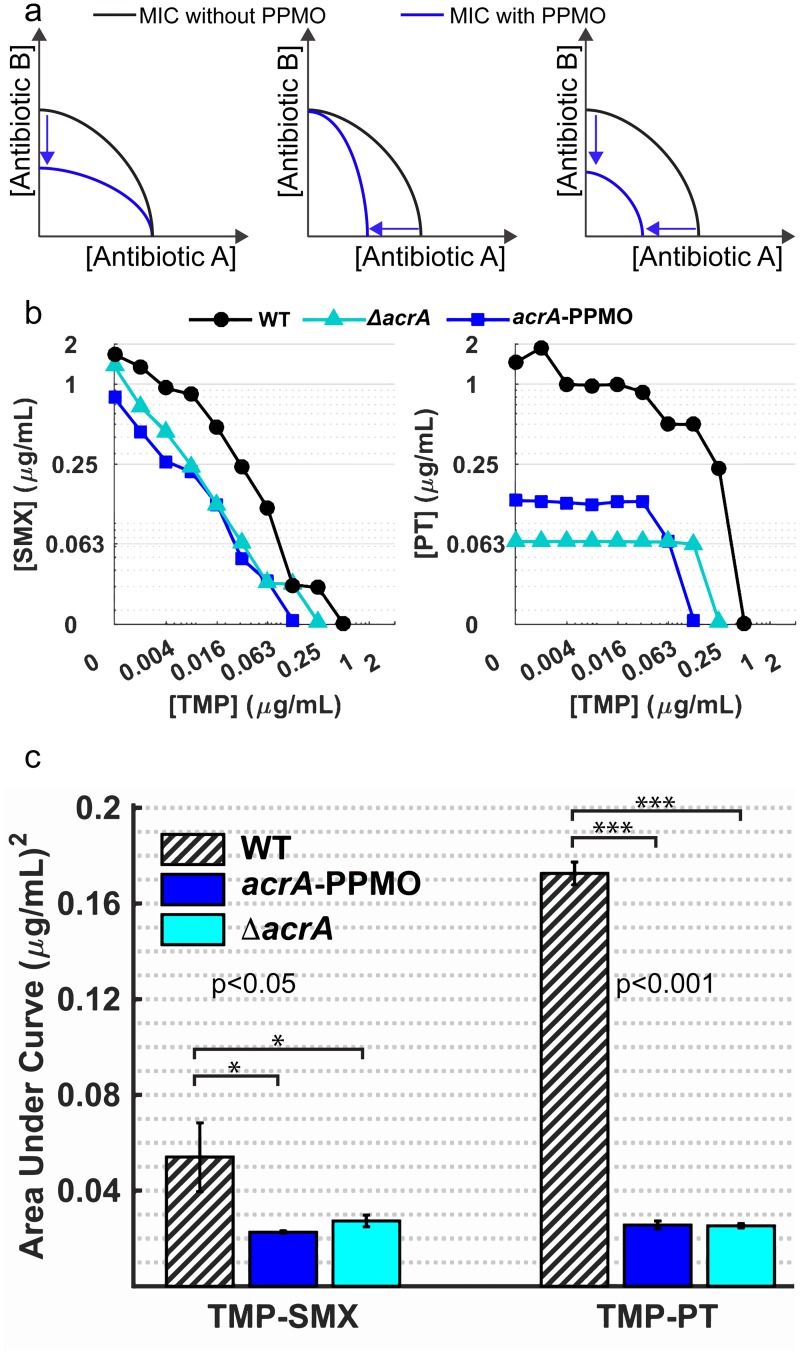
Targeting resistance genes with *acrA*-PPMO increases efficacy of antibiotic combinations and even makes the use of antagonistic antibiotic pairs possible. (A) Conceptual representation of the possible effects of efflux inhibition on the use of antibiotics pairs. Blue and black lines represent the MIC lines in two-dimensional gradients of drug pairs for bacteria with and without *acrA*-PPMO, respectively. The left panel represents an increase in susceptibility to antibiotic B, the middle panel represents an increase to antibiotic A, and the right panel represents an increase to both antibiotics. (B) MIC lines determined in two-dimensional gradients of (left) trimethoprim-sulfamethoxazole and (right) trimethoprim-piperacillin/tazobactam for wild type *E*. *coli* (black line), with 10 μM *acrA*-PPMO (blue line), or the *acrA* deletion mutant (cyan line). (C) Bar graphs demonstrating the efficacy of antibiotic combinations shown in (B). Area under the MIC curves in (B) are significantly reduced relative to the wild type *E*. *coli* (bars with black diagonal lines) for both antibiotic combinations when 10 μM *acrA*-PPMO (blue bars) is used or *acrA* (cyan bars) is physically deleted. All measurements were done in triplicate, and *p*-values for significance were calculated with Student’s *t*-test.

One strategy often employed for treatment of severe bacterial infections is the combined use of two or more antibiotics with different mechanisms of action [29]. Particularly, the use of antibiotic pairs that display synergy is considered to be advantageous in clinical practice [30,31]. One risk of this approach is that several synergistic antibiotic pairs may promote evolution of multidrug resistance if they have overlapping resistance mechanisms [5,32,33]. Conversely, several antibiotic pairs that are less likely to promote resistance cannot be used in combination due to antagonistic drug—drug interactions [5,30]. Therefore, strategies that could rescue the use of antagonistic drug combinations could be of significant benefit [34]. Successfully enhancing antibiotic susceptibility by blocking efflux activity has three potential outcomes in antibiotic combination therapies (Fig 5A). It could increase susceptibility to either antibiotic independently (Fig 5A, left and middle), or it could increase susceptibility to both drugs simultaneously (Fig 5A, right). We tested the *acrA*-PPMO together with antibiotic pairs to see if we could improve their antimicrobial efficacy. Here, we demonstrate that sensitizing bacteria against antibiotics by targeting *acrA* with the *acrA*-PPMO can increase sensitivity to both synergistic and antagonistic pairs. We quantified pairwise interactions between trimethoprim and sulfamethoxazole versus trimethoprim and piperacillin-tazobactam, in the presence and absence of *acrA*-PPMO (Fig 5B). Trimethoprim and sulfamethoxazole are antifolate antibiotics that block the activity of dihydrofolate reductase and dihydropteroate synthase, respectively. Trimethoprim is often used together with sulfamethoxazole due to their synergistic interaction [30]. Conversely, using trimethoprim with piperacillin-tazobactam could be problematic since these two drugs were previously reported to antagonize each other’s activities [30]. We created two-dimensional gradients of trimethoprim-sulfamethoxazole (Fig 5B, left) or trimethoprim-piperacillin/tazobactam (Fig 5B, right) and determined MIC values for the wild-type *E*. *coli* in the presence (Fig 5B, blue lines) and absence of *acrA*-PPMO (Fig 5B, black lines) or with the *acrA* deletion (Fig 5B, cyan lines). We compared the enhancement of drug combinations by *acrA*-PPMO by integrating the AUC of the resulting MIC curves (Fig 5C). We found that *acrA*-PPMO increases the efficacy of both synergistic and antagonistic pairs by nearly 5-fold and 15-fold for trimethoprim-sulfamethoxazole (Fig 5C, left) and trimethoprim-piperacillin/tazobactam combinations (Fig 5C, right), respectively. This observation clearly indicates that even though trimethoprim and piperacillin-tazobactam have antagonistic interactions, *acrA*-PPMO significantly (*p* < 0.001) increases the efficacy of the trimethoprim-piperacillin-tazobactam combination in *E*. *coli*. This finding has the potential of making the trimethoprim-piperacillin-tazobactam combination a promising candidate for treating infections since trimethoprim and piperacillin-tazobactam have independent resistance mechanisms that make the emergence of cross-resistance less likely [5,7]. PPMO treatment did not change the shape of the MIC curves for both of the trimethoprim-sulfamethoxazole (Fig 5B, left) and trimethoprim-piperacillin/tazobactam (Fig 5B, right) combinations, but rather rescaled the MIC curves towards the origin compared to the wild type (Fig 5B), as was previously demonstrated for other antibiotics by Chait et al. [34]. We conclude that the *acrA*-PPMO did not affect the drug interaction mechanisms, but rather, the decreased efflux of both antibiotic compounds resulted in increased effective antibiotic concentrations inside bacterial cells.

## Discussion

In this study, we demonstrate that we can strategically induce antibiotic hypersensitivity in pathogenic bacteria by targeting the genes that encode for the AcrAB-TolC efflux system with PPMOs. Antibiotic molecules that traverse the bacterial membrane can therefore remain intracellular for longer time periods leading to increased antibiotic activity. This could have also been achieved by using efflux pump inhibitor molecules, such as phenyl-arginine-β-naphthylamide, that block AcrAB-TolC activity [35–39]. However, efflux pump inhibitors are known to have significant toxicities and currently have limited use as therapeutic agents [35,36]. The method we introduced for inducing antibiotic hypersensitivity with the use of PPMOs does not exhibit cytotoxicity in the human cell line we tested and does not require editing the genome of the targeted bacteria in the human host. There are several advantages associated with increasing antibiotic susceptibility of pathogens by using PPMOs. First, though this is an in vitro demonstration, it suggests the potential of using lower doses of existing antibiotics, which may lead to fewer adverse effects of those agents. Second, being able to sensitize a specific bacterial pathogen and inhibit its growth by lower antibiotic doses could have the potential to minimally perturb beneficial members of healthy human microbiota. Third, by using PPMOs, we may have the opportunity to use several drugs for treating infections against which they are normally not effective, such as oxacillin against gram-negative bacteria (Fig 3B and S4 Fig). Finally, increasing antibiotic efficacy with PPMOs may change the way we typically design combinatorial antibiotic therapies: by using PPMOs to minimize the antibiotic doses necessary in antibiotic combinations, we may be able to use antibiotic pairs even if the two drugs somewhat dampen each other’s inhibitory effects. However, pharmacokinetics of PPMOs and potential antibiotic combinations should be considered when designing combination therapies for maximum antimicrobial activity [40]. In addition to the benefits described above, the sequence-specificity of PPMOs allows for the ability to target a single genus, or multiple genera, if the PMO target sequence is conserved (Fig 3C). As we have previously reported in *Burkholderia*, significant reductions in efficacy (> 8-fold) can occur with even single base mismatches in the PMO sequence [11]. Additionally, Tilley et al. demonstrated that four silent mutations were sufficient to render a targeted PMO ineffective in *E*. *coli* [41]. However, the relationship between mismatches, including number and where they occur spatially in the oligomer sequence, and impact on efficacy have not been thoroughly described and warrant future study.

We conclude that targeting resistance genes with PPMOs is a plausible strategy to increase antibiotic susceptibility in pathogenic bacteria. Further studies are needed to extend our in vitro experiments to animal models of infection to bridge the gap between our in vitro experiments and translational studies in humans. Utilizing sequence-specific PPMOs that do not have antimicrobial activity when used alone has the potential advantage of avoiding classic selection pressure exhibited by traditional antimicrobials. Importantly, acquired bacterial resistance to PPMOs has thus far only been described in the context of PPMOs designed against essential genes and was found to be related to the peptide moiety and not the oligomer sequence [42]. Attachment of a different peptide to the same oligomer was able to rescue PPMO activity, indicating possible paths towards dealing with the development of resistance. Given the narrow pipeline for new antibiotics and the increasingly urgent worldwide problem of antibiotic resistance, innovative therapeutic approaches such as utilizing PPMOs could serve an important medical need in the future. Future studies will be conducted to systematically test the strategies we propose in this paper in preclinical in vivo models to bridge the gap between in vitro experiments and human studies.

## Materials and Methods

### Growth Media and Strains

Bacterial cells were grown at 37°C in M9 minimal medium (248510, Difco) supplemented with 0.4% glucose (50-99-7, Fisher Scientific) and 0.2% amicase (82514, Sigma), if not stated otherwise. All *E*. *coli* strains were wild type K-12 derivatives of the parent strain BW25113. The deletion strains were generated using the Keio Collection [43]; Δ*acrA*, Δ*acrB*, Δ*cmr*, Δ*emrB*, Δ*marB*, Δ*ompF*, Δ*tolC* were obtained from the *E*. *coli* Genetic Stock Center with stock codes 11843, 8609, 8865, 10099, 9314, 8925, 11430, respectively. These strains were used for making P1 lysates and generating mutant strains with multiple gene deletions by P1 phage transduction [44]. Kanamycin resistance marker genes were removed after every cloning step [43,45].

### Antibiotic Compounds

The antibiotics used in this study are: Ampicillin (A1593, Sigma-Aldrich), Carbenicillin (C3416, Sigma-Aldrich), Cefotaxime (454950050, Acros Organics), Cefoxitin (C4786, Sigma-Aldrich), Chloramphenicol (C0378, Sigma-Aldrich), Ciprofloxacin (F17850, Sigma), Clindamycin (21462-39-5, RPI), Doxycycline (D9891, Sigma-Aldrich), Erythromycin (E5389, Sigma-Aldrich), Fusidic acid (F0881, Sigma-Aldrich), Gentamycin (PRX1002-Premier Pro RX), Kanamycin (60616, Sigma-Aldrich), Levofloxacin (28266, Sigma-Aldrich), Lomefloxacin (L2906, Sigma), Meropenem (NDC6332350720, Fresenius Kabi LLC), Nitrofurantoin (N7878, Sigma-Aldrich), Oxacillin (NDC25021-162-24, Sagent Pharmaceuticals), Penicillin (P8396 Sigma-Aldrich), Piperacillin/tazobactam (NDC60505-0688-4, Apotex Corp.), Rifampicin (R3501, Sigma-Aldrich), Spectinomycin (85555, Sigma-Aldrich), Spiramycin (S9132, Sigma-Aldrich), Sulfamonomethoxine (32091, FLUKA), Tetracycline (87128, Sigma-Aldrich), Tobramycin (T4014, Sigma-Aldrich), Trimethoprim (T7883, Sigma-Aldrich), Vancomycin (NDC67457-340-00, Mylan). All antibiotic solutions were prepared by following manufacturers’ instructions.

### PPMOs Used for Targeting Efflux Genes

PMOs were synthesized as previously described [46]. The cell-penetrating peptide (RXR)_4_XB, where R is arginine, X is aminohexanoic acid, and B is beta-alanine, was synthesized using standard FMOC chemistry and purified to >95% purity at CPC Scientific (Sunnyvale, CA) and used without further purification. The peptide was conjugated to the nitrogen of a piperadine ring at the 5′-terminus of the PMO. First, a C-terminally reactive peptide-benzotriazolyl ester was prepared by dissolving the peptide acid with O-(Benzotriazol-1-yl)-N,N,N',N'-tetramethyluronium tetrafluoroborate (TBTU) in 1-methyl-2-pyrrolidinone (NMP). The concentration of the peptide was 50 mM. Diisopropylethylamine (DIEA) was added to the peptide solution at molar ratios of peptide acid:TBTU:DIEA of 1.0:1.5:1.5, respectively. Immediately after the addition of DIEA, the peptide solution was added to a DMSO solution containing the PMO (20 mM) at a 1:0.8 molar ratio. After stirring at 25°C for 3 h, the reaction was stopped by adding a 4-fold volumetric excess of water. 1 M H_3_PO_4_ was added to crude conjugated PMO in 50 μL aliquots until pH 3 was reached. After stirring at 25°C for 30 min, the reaction was neutralized by adding 1 M Na_2_HPO_4_ in 100 μL aliquots until pH 7 was reached. The resulting solution was loaded onto a Source 30s (Sigma, St. Louis, MO) column. The unconjugated PMO and other reaction products were purified by elution with a 1.5 M Guanidine-HCl solution in 20 mM NaH_2_PO_4_ with 25% MeCN in Milli-Q water at pH 6.5 from 0%–50% over 12 columns volumes. Fractions were selected and pooled based on UV absorbance. Pooled fractions were diluted by adding a 5-fold volumetric excess of water and the conjugate/salt solution was then loaded onto a SPE column (Amberchrom CG300M, Dow Chemicals, MI), which was subsequently washed three times with two-column volumes of water to remove salt. Finally, the (RXR)_4_XB—PMO conjugate was eluted off the SPE column with two-column volumes of 50% MeCN and lyophilized. The final products were analyzed by matrix-assisted laser desorption ionization time of flight mass spectrometry and HPLC. The purities of the final products were >85%. The nucleotide sequence for the control-PPMO is ATCGTTGCATC, for *acrA*-PPMO is GTTCATATGTA, for *acrB*-PPMO is TAGGCATGTCT, and for *tolC*-PPMO is TTCATTTGCAT.

### MIC Determinations

Master plates of each bacterial strain were prepared in a 96-well plate format using overnight cultures in ~15% glycerol (~5 x 10^8^ CFU/mL) and stored at −80°C. The master plates were thawed prior to experiments and then used to inoculate the antibiotic plates with a pinner (VP Scientific, VP409), which transfers ~5 x 10^4^ CFUs into each well containing ~200 μL of growth media. MIC values were determined using either end-point (final OD600) analysis or calculating the AUC [47]. Briefly, for end-point MIC determination (Figs 1 and 3 and S2 and S3 Figs), the 96-well plates were incubated for 22 h in a shaker operated at 37°C and then OD600 of each plate was measured using a plate reader (Infinite M200 Pro, Tecan). For each strain and antibiotic pair, the MIC value was defined as the lowest antibiotic concentration at which the final OD600 was below ~0.04 after background correction, which is slightly above the lower detection limit of our plate reader. Preliminary experiments were conducted with four replicates for clindamycin and fusidic acid. The remaining antibiotics were run twice with biological replicates. A Pearson correlation coefficient test was used to confirm the repeatability of the measurements and the *p*-values for MIC reduction significance were calculated using Wilcoxon rank sum test. For MIC determination using AUC values, plates were incubated under similar environmental conditions, but in an automated robotic system so that OD600 of growing cultures were recorded as a function of time (Fig 2D and 2E). Linear interpolations of the resulting growth curves (OD600 versus time, Fig 2D) were then integrated to calculate the AUC as a metric for growth using a custom MATLAB code (r2016a, MathWorks). MIC values were defined as the concentration of antibiotic where the AUC was reduced by at least 95% compared to the AUC without antibiotic. Although both methods gave qualitatively similar results, we used the AUC method whenever possible because it is more robust to experimental noise [47].

### Time-Kill Assay of Piperacillin/Tazobactam with AcrA PPMO

*E*. *coli* BW25113, *K*. *pneumoniae* F45153 (clinical urine isolate), *S*. *enterica enterica* serovar Typhimurium 14028S (a generous gift from Dr. Sebastian Winter, UTSW Medical Center), *A*. *baumannii* AYE, *P*. *aeruginosa* PAO1, and *B*. *cenocepacia* complex K56-2 (cystic fibrosis clinical isolate) were grown overnight in cation-adjusted Mueller-Hinton II broth (MHII, Becton, Dickinson and Co., Sparks, MD) at 37°C, 220 rpm. Cultures were diluted to 5 x 10^5^ CFU/mL in fresh MHII and incubated with 2-fold dilutions of piperacillin/tazobactam alone or in combination with 10 μM control-PPMO or acrA-PPMO in a 96-well plate and incubated for 18 h at 37°C, 220 rpm. Growth controls included H_2_O, 10 μM control-PPMO, and 10 μM acrA-PPMO alone. The MIC was evaluated at 18 h using the final optical density values as described above; for reference, the MIC values were 4, 2, 2, 128, 4, and 64 μg/mL for *E*. *coli* BW25113, *K*. *pneumoniae* F45153, *S*. *enterica* 14028S, *P*. *aeruginosa* PAO1, *A*. *baumannii* AYE, and *B*. *cenocepacia* K56-2, respectively. Growth controls and wells at 1-, 0.5-, and 0.25-fold the MIC of piperacillin/tazobactam alone or in combination with PPMO were serially diluted in PBS and plated on trypticase soy agar with 5% sheep blood (Remel, Lenexa, KS) for CFU enumeration. Experiments were performed with at least four replicates. Experiments with some of the strains were carried out with six or nine replicates, because the dose-response curves of piperacillin-tazobactam are very steep in general and it is difficult to precisely measure MIC values with 2-fold dilutions.

### Quantifying AcrA Expression with Western Blots

Western blots measuring AcrA levels after PPMO addition (1 to 12 μM) were performed following standard procedures using an AcrA antibody (1:30,000; generous gift from Dr. Helen I. Zgurskaya, University of Oklahoma) and cAMP receptor protein antibody (1:1,000; BioLegend: 664304). *E*. *coli* cells (BW25113) were grown overnight, and final OD600 was adjusted to unity. These cells were then diluted by 10^4^ fold in 5 mL of M9 minimal media (supplemented with 0.4% glucose and 0.2% amicase) and grown for 6 h at 37°C (220 rpm) in the presence of increasing *acrA*-PPMO concentrations (1–12 μM final concentration). Cells were then washed three times with cold PBS buffer (pH 7.4), and bacterial pellets were lysed in 1X Laemmli sample buffer (5 mL/O.D.). Equivalent amounts of the cell lysates (10 μL of the above sample) from each set were electrophoresed in a 4%–15% precast polyacrylamide gel (561081; BIO-RAD), and western blotting was performed following standard procedures. IR-labeled secondary antibodies (IRDye 800CW (926–32213) and IRDye 680RD (925–68072); Li-COR) were used for detection. AcrA protein amount was quantified using an ODYSSEY infrared imaging system (LI-COR).

### Efflux Inhibition Assay

*E*. *coli* (BW25113) and the *acrA* gene deletion *E*. *coli* strain were grown overnight, and final OD600 was adjusted to unity. The cells were then diluted by 10^3^-fold in M9 minimal media (with 0.4% glucose and 0.2% amicase) and grown for 6 h at 37°C (100 rpm) in the presence of 0, 2, and 10 μM acrA-PPMO concentrations until the OD600 reached ~0.25. The cells were washed twice in PBS buffer (pH 7.4) and were diluted to a final OD600 of 0.2 and 0.4 (three replicates for each cell density). Hoechst 33342 dye (Thermo Fisher, 62249) was then added to final dye concentration of 10 μM in a 96-well plate. Fluorescence and OD600 measurements were immediately recorded every ~75 s for 10 h (S6A Fig). Fluorescence of Hoechst 33342 was measured by excitation at 361 nm and emission centered at 497 nm. Fluorescence values were corrected by subtracting fluorescence of PPMOs mixed with Hoechst 33342 dye in the absence of bacterial cells. This step was crucial, since *acrA*-PPMO produced significant fluorescence due to its AT-rich sequence yielding high fluorescence quantum yield for the Hoechst 33342 dye. Fluorescence intensity (FI) values were normalized with the optical density of bacterial cultures. Fluorescence accumulation rates were calculated by fitting a line to the normalized FI values that were recorded within the first 5–10 min of the experiment when FI linearly increases (S6B and S6C Fig). Final FI levels were calculated by averaging fluorescence within a time window where fluorescence and OD values remain constant (S6D Fig).

### Cloning and Expression of Rescue Plasmids Carrying Efflux Genes

For reversing the antibiotic sensitivity phenotype of *E*. *coli* with efflux gene deletions, we cloned *acrA*, *acrB*, and *tolC* genes into the arabinose inducible pJMK001 plasmid (Addgene) and introduced them into gene deletion strains. The efflux pump genes (*acrA*, *acrB*, and t*olC*) were PCR amplified from the wild type (BW25113) *E*. *coli* strain using the following primer sets (*acrA*-forward: CATGCCATGGGGATGAACAAAAACAGAGGGTTTACG, *acrA*-reverse: AGCTTTGTTTAAACTTAAGACTTGGACTGTTCAGGCTG); (*acrB*-forward: CATCAGTCATGATGCCTAATTTCTTTATCGATCG, *acrB*-reverse: AGCTTTGTTTAAACTCAATGATGATCGACAGTATG) and (*tolC*-forward: CATGCCATGGGGATGAAGAAATTGCTCCCCATTC; *tolC*-reverse: AGCTTTGTTTAAACTCAGTTACGGAAAGGGTTATGA). These fragments were cloned into pJMK001 after restriction digestion (NcoI and PmeI) followed by ligation. These plasmids were then transformed into *E*. *coli* strains that had relevant gene deletions. For expression of the efflux genes, bacterial cultures with and without plasmids were grown in the presence of 0.2% arabinose in M9 minimal medium.

## Supporting Information

S1 Fig Single and combinatorial gene deletions were verified by PCR amplification of the chromosomal regions that span the genes of interest. The approximate PCR product sizes are 3 kb, 1.2 kb, 200 b, 1.5 kb, and 1 kb for *acrB* (ΔA), *cmr* (ΔC), *marB* (ΔM), *emrB* (ΔE), *and ompF* (ΔO), respectively. As can be seen from the gel images, we also deleted *marA* (ΔR) and *tolC* (ΔT) alone and in combination with select genes. We did not perform further deletions in combination with *marA* or *tolC* since the phenotypic effect of *marA* deletion did not have a significant effect on antibiotic resistance, and *tolC* deletion was indistinguishable from the deletion of *acrB*.
(TIF)

S2 Fig (A) Heat map showing the normalized MIC values of every gene deletion strain, for the 27 tested antibiotic compounds. All measurements were done in at least duplicate. Measurements with the wild-type strain were done with eight replicates. Measurements with clindamycin and fusidic acid were done with four replicates. Statistically significant (*p* < 0.05) changes in MIC compared to the wild type strain are depicted colorimetrically, with red representing decreases in efficacy, blue representing increases in efficacy, and white representing nonsignificant changes in efficacy. Intensities of the blue and red colors indicate the magnitude of efficacy changes. The actual MIC values can be found in S1 Table. (B) MIC measurements of the gene deletion strains across duplicates were highly reproducible. The Pearson correlation coefficient between MIC values for the replicate measurements is 0.97 (*p* < 0.001), demonstrating the reproducibility of our measurements. Mean value for the ratios between MIC_replicate1_ and MIC_replicate2_ measurements is 1.07 ± 0.55 (mean ± standard deviation).
(TIF)

S3 Fig Deleting any of the *acrA*, *acrB*, and *tolC* genes reduces the erythromycin MIC of *E*. *coli*, and reintroducing these genes on plasmids (pJMK001) reverses the decreased MIC (*p* < 0.001). Measurements were done in six replicates.
(TIF)

S4 Fig (A–C) Silencing *acrA*, *acrB*, or *tolC* increases susceptibility of *E*. *coli* against several antibiotics. Dose-response curves as a function of drug concentration for the wild type without PPMO (black lines), with 10 μM control-PPMO (grey lines), with 10 μM *acrA*-PPMO (top panel, blue lines), *E*. *coli* with *acrA* deletion (top panel, cyan lines), with 10 μM *acrB*-PPMO (middle panel, magenta lines), *E*. *coli* with *acrB* deletion (middle panel, pink lines), with 10 μM *tolC*-PPMO (bottom panel, dark green lines), and *E*. *coli* with *tolC* deletion (bottom panel, light green lines). (D) MIC measurements across duplicates (shown in A–C) were highly reproducible. The Pearson correlation coefficient between MIC values for the replicate measurements is ~0.99 (*p* < 0.001), demonstrating the reproducibility of our measurements. Mean value for the ratios between MIC_replicate1_ and MIC_replicate2_ measurements is 1.06 ± 0.28 (mean ± standard deviation).
(TIF)

S5 Fig Western blots for AcrA and the CRP (cAMP receptor protein) demonstrate that control PPMO does not reduce AcrA protein levels compared to the wild type *E*. *coli*. On the other hand, use of *acrA*-PPMO (2 and 10 μM) and deletion of *acrA* significantly reduces AcrA protein levels.
(TIF)

S6 Fig Silencing *acrA* with acrA-PPMO (2 and 10 μM) and deletion of the *acrA* gene decrease Hoechst 33342 efflux. Hoechst 33342, a DNA-intercalating dye, is a substrate of the AcrAB-TolC system. We measured the influx of Hoechst 33342 into *E*. *coli* cells by recording fluorescence and OD600 values of bacterial cultures. Note that influx of the dye molecules reflects the difference between concentration-dependent dye influx and AcrAB-TolC-related dye efflux. All measurements were done in six replicates, and fluorescence values were normalized by the OD600 of the corresponding cultures. (A) Fluorescence created by the addition of 10 μM Hoechst 33342 dye was recorded for 10 h. All values are normalized with respect to the mean FI values of the untreated wild-type *E*. *coli* cells (black line) recorded during the last 5 h when fluorescence remain unchanged. Fluorescence of wild type *E*. *coli* cells treated with the control-PPMO (gray dashed line) was similar to fluorescence of untreated wild type *E*. *coli* cells (black line). Fluorescence of *acrA*-PPMO-treated cells (2 μM *acrA*-PPMO: blue dashed line; and 10 μM *acrA*-PPMO: blue continuous line) and the *acrA* deletion cells (cyan line) reached slightly higher fluorescence levels at a higher initial rate. (B) Fluorescence measurements within the first 20 min (shown in A) demonstrate that the net influx rate of the Hoechst 33342 dye increases if *acrA* is deleted (cyan lines) or silenced (blue lines). This increase reflects the decreased efflux of the Hoechst 33342 dye by AcrAB-TolC complex. (C) Bar graph showing normalized mean fluorescence accumulation rates measured within first 5 min (B) after dye addition. Error bars represent standard deviations of six measurements. Accumulation rate increases by nearly 2.5 times (*p* < 0.001) when *acrA* is silenced or deleted. (D) Bar graph showing normalized mean final fluorescence values measured within the last 5 h (A) after dye addition. Error bars represent standard deviations of six measurements. Final fluorescence increases by nearly 1.3 times (*p* < 0.01) when *acrA* is silenced or deleted.
(TIF)

S1 Table MIC values used to generate S2 Fig are provided. (XLSX)

S2 Table MIC values used to generate S4D Fig are provided. (XLSX)

S3 Table The numeric data to generate Figs 1B and 1C, 2E, 3, 4 and 5B and 5C, S3, S4 and S6 Figs are provided. (XLSX)

## Abbreviations

AUC: area under the curve
CFU: colony forming unit
CRP: cAMP receptor protein
DIEA: diisopropylethylamine
FI: fluorescence intensity
MIC: minimum inhibitory concentration
NMP: 1-methyl-2-pyrrolidinone
OD600: optical density at 600 nm
PMO: phosphorodiamidate morpholino oligomer
PPMO: peptide-conjugated phosphorodiamidate morpholino oligomer
TBTU: O-(Benzotriazol-1-yl)-N,N,N',N'-tetramethyluronium tetrafluoroborate
