# Supplementary figures and images for: Sequence-Specific Targeting of Bacterial Resistance Genes Increases Antibiotic Efficacy
Source: PLoS Biol. 2016 Sep 15;14(9):e1002552. doi: 10.1371/journal.pbio.1002552 (PMC5025249; doi:10.1371/journal.pbio.1002552)

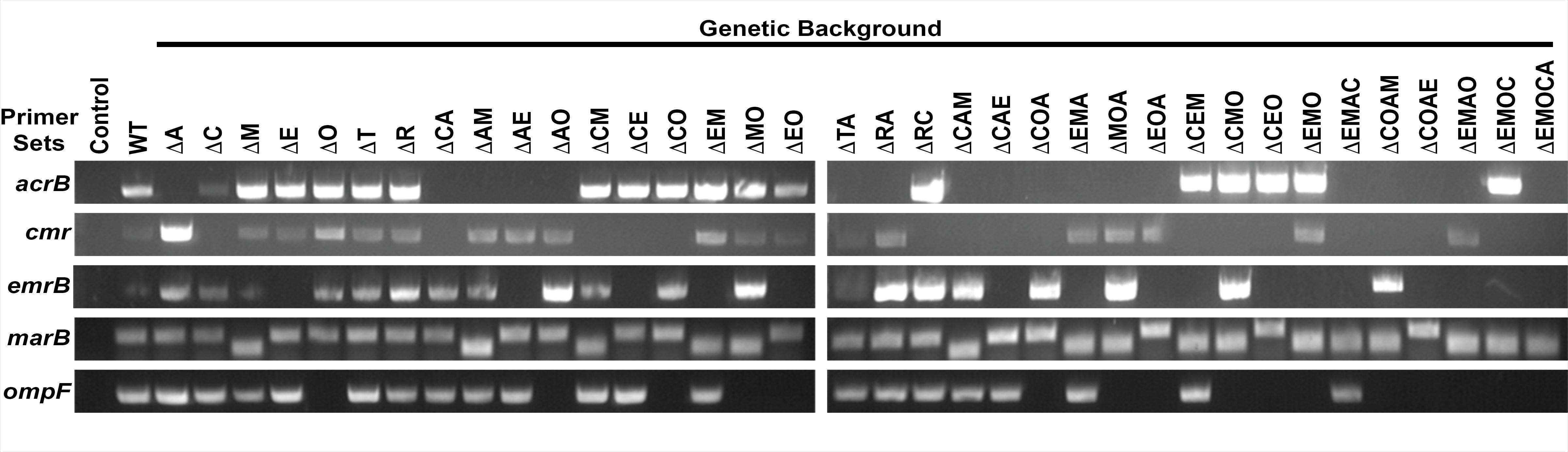

Supplement: S1 Fig — The approximate PCR product sizes are 3 kb, 1.2 kb, 200 b, 1.5 kb, and 1 kb for acrB (ΔA), cmr (ΔC), marB (ΔM), emrB (ΔE), and ompF (ΔO), respectively. As can be seen from the gel images, we also deleted marA (ΔR) and tolC (ΔT) alone and in combination with select genes. We did not perform further deletions in combination with marA or tolC since the phenotypic effect of marA deletion did not have a significant effect on antibiotic resistance, and tolC deletion was indistinguishable from the deletion of acrB. (TIF) [file pbio.1002552.s001.tif]

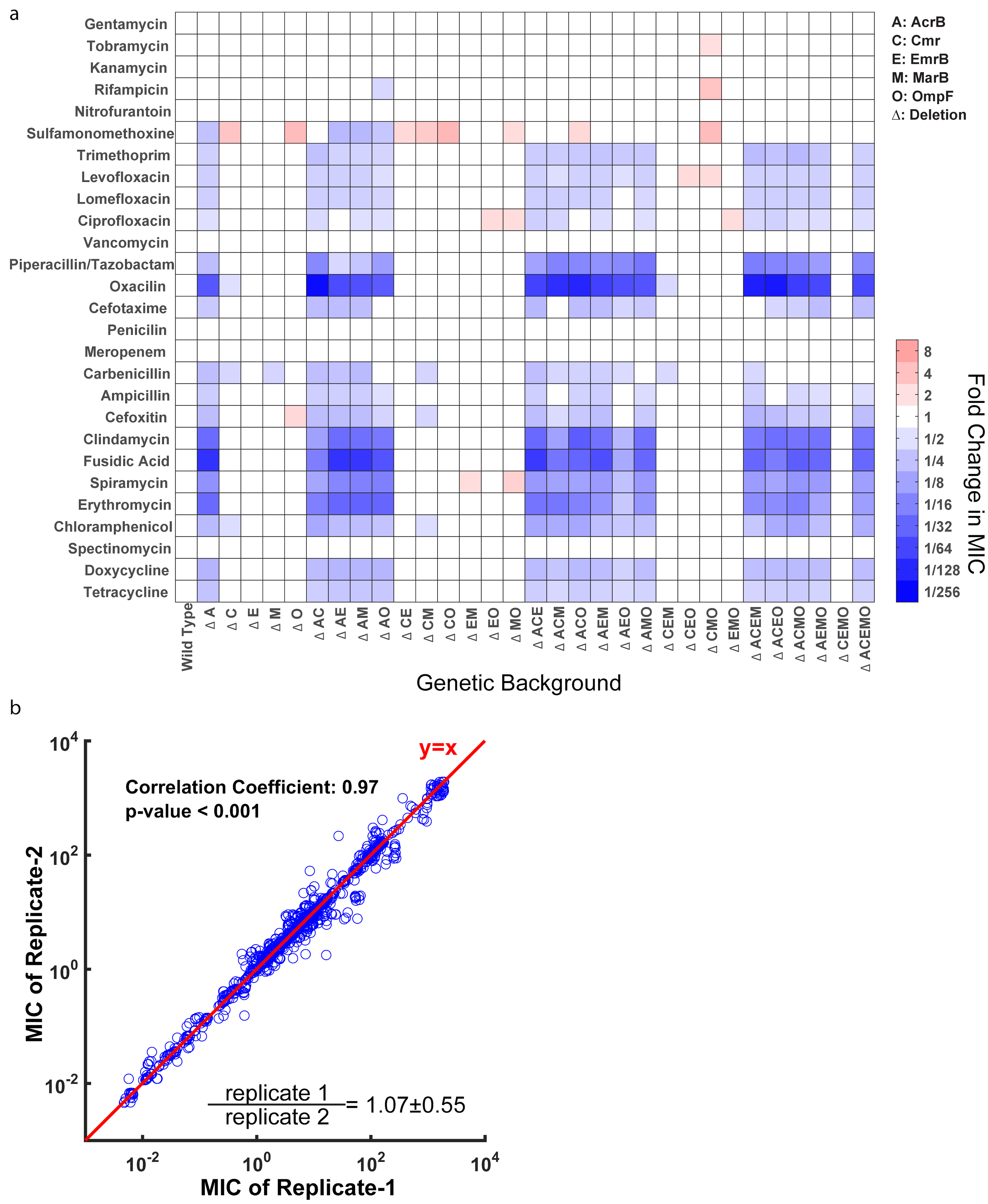

Supplement: S2 Fig — (A) Heat map showing the normalized MIC values of every gene deletion strain, for the 27 tested antibiotic compounds. All measurements were done in at least duplicate. Measurements with the wild-type strain were done with eight replicates. Measurements with clindamycin and fusidic acid were done with four replicates. Statistically significant (p < 0.05) changes in MIC compared to the wild type strain are depicted colorimetrically, with red representing decreases in efficacy, blue representing increases in efficacy, and white representing nonsignificant changes in efficacy. Intensities of the blue and red colors indicate the magnitude of efficacy changes. The actual MIC values can be found in S1 Table. (B) MIC measurements of the gene deletion strains across duplicates were highly reproducible. The Pearson correlation coefficient between MIC values for the replicate measurements is 0.97 (p < 0.001), demonstrating the reproducibility of our measurements. Mean value for the ratios between MICreplicate1 and MICreplicate2 measurements is 1.07 ± 0.55 (mean ± standard deviation). (TIF) [file pbio.1002552.s002.tif]

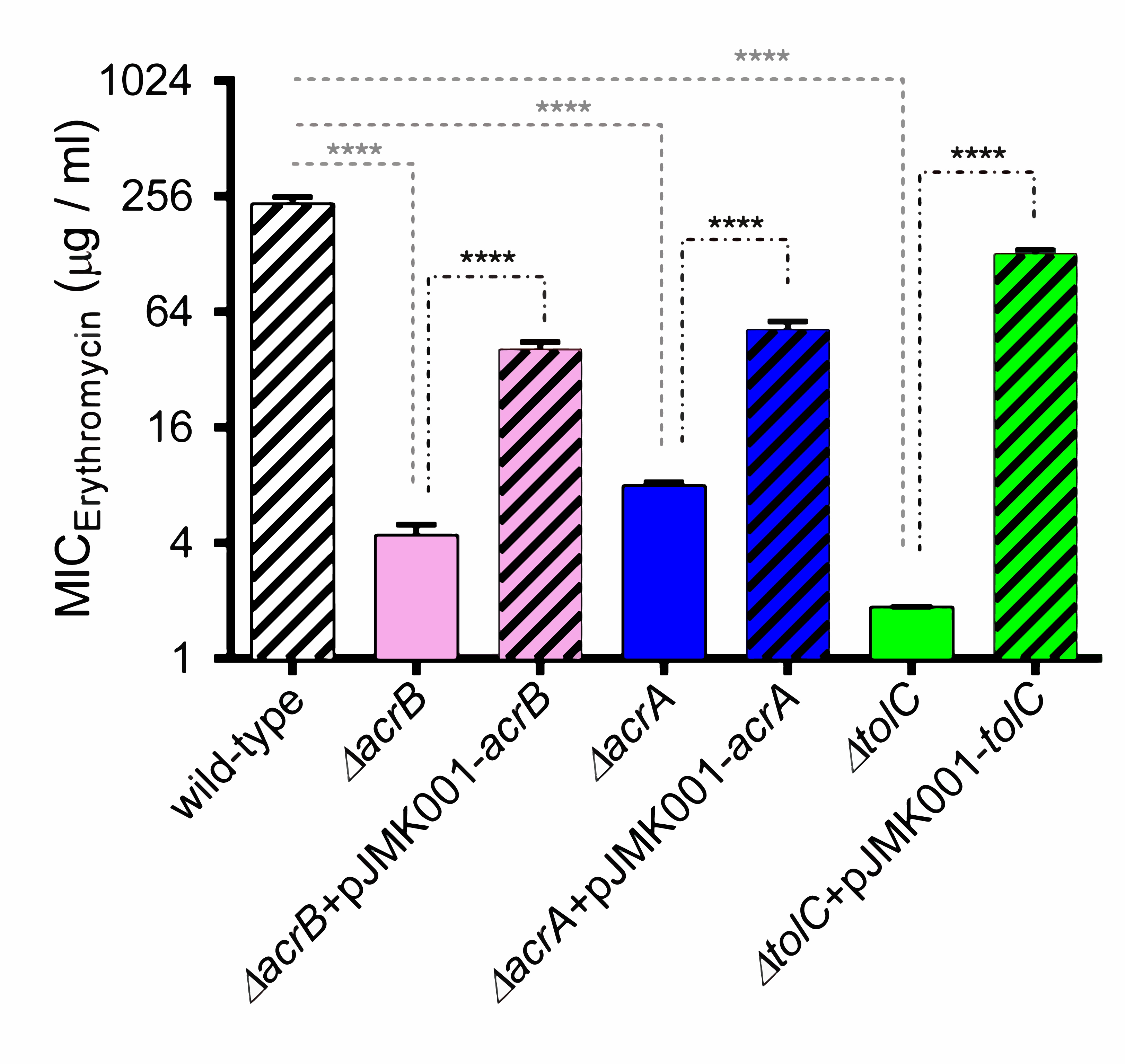

Supplement: S3 Fig — Measurements were done in six replicates. (TIF) [file pbio.1002552.s003.tif]

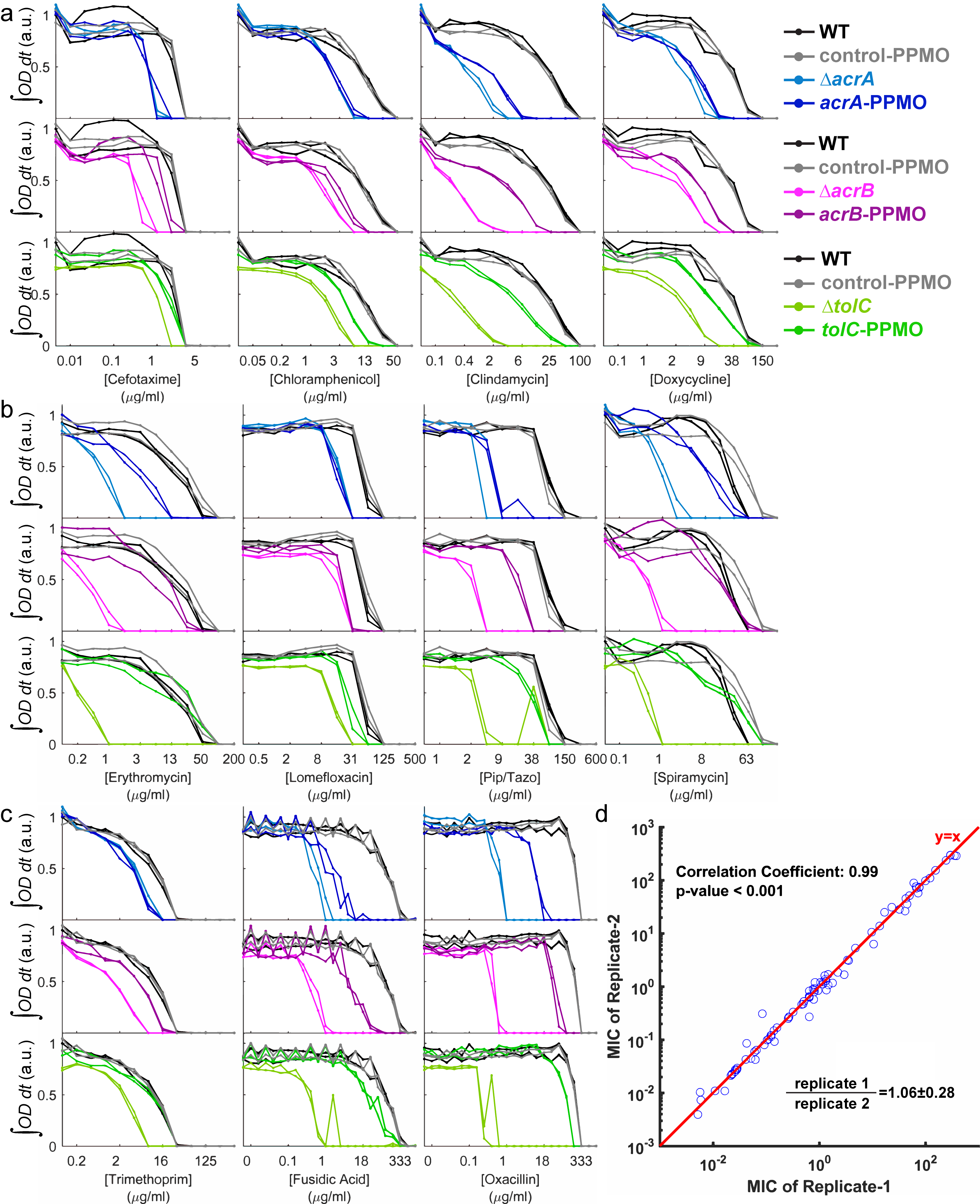

Supplement: S4 Fig — (A–C) Silencing acrA, acrB, or tolC increases susceptibility of E. coli against several antibiotics. Dose-response curves as a function of drug concentration for the wild type without PPMO (black lines), with 10 μM control-PPMO (grey lines), with 10 μM acrA-PPMO (top panel, blue lines), E. coli with acrA deletion (top panel, cyan lines), with 10 μM acrB-PPMO (middle panel, magenta lines), E. coli with acrB deletion (middle panel, pink lines), with 10 μM tolC-PPMO (bottom panel, dark green lines), and E. coli with tolC deletion (bottom panel, light green lines). (D) MIC measurements across duplicates (shown in A–C) were highly reproducible. The Pearson correlation coefficient between MIC values for the replicate measurements is ~0.99 (p < 0.001), demonstrating the reproducibility of our measurements. Mean value for the ratios between MICreplicate1 and MICreplicate2 measurements is 1.06 ± 0.28 (mean ± standard deviation). (TIF) [file pbio.1002552.s004.tif]

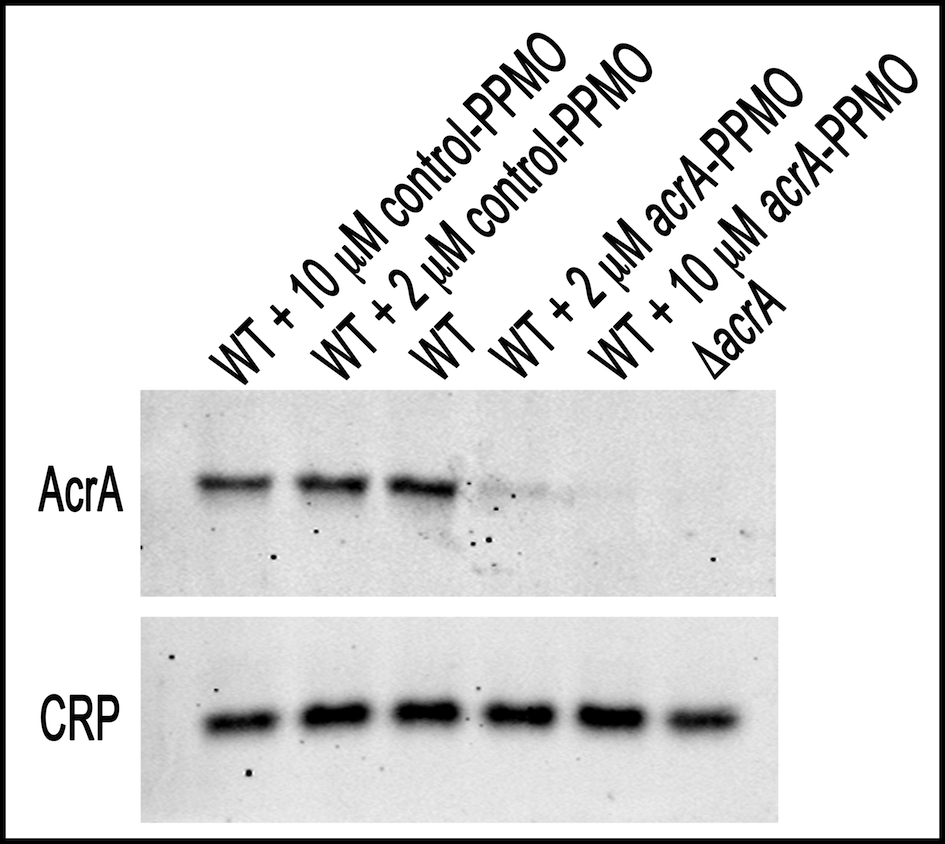

Supplement: S5 Fig — On the other hand, use of acrA-PPMO (2 and 10 μM) and deletion of acrA significantly reduces AcrA protein levels. (TIF) [file pbio.1002552.s005.tif]

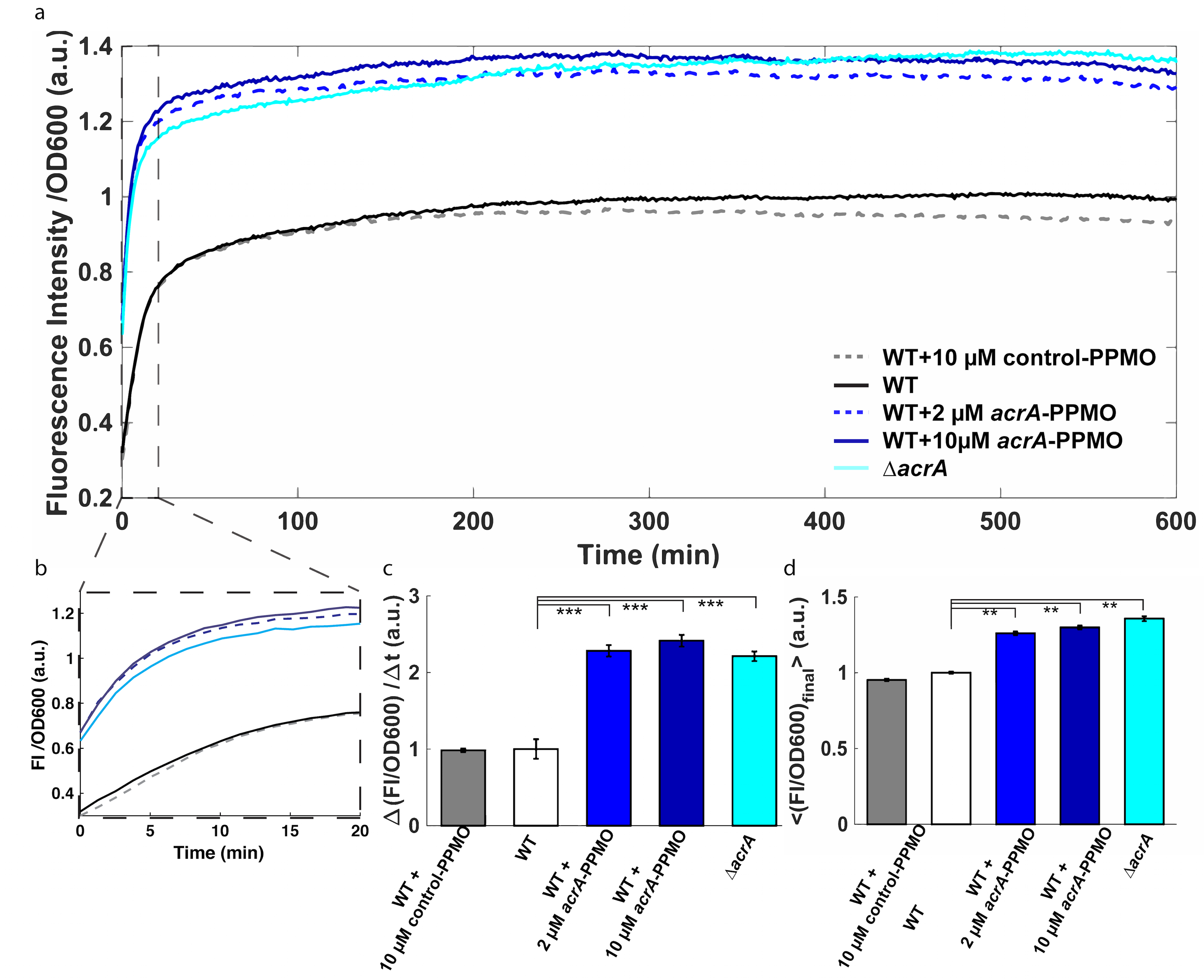

Supplement: S6 Fig — Hoechst 33342, a DNA-intercalating dye, is a substrate of the AcrAB-TolC system. We measured the influx of Hoechst 33342 into E. coli cells by recording fluorescence and OD600 values of bacterial cultures. Note that influx of the dye molecules reflects the difference between concentration-dependent dye influx and AcrAB-TolC-related dye efflux. All measurements were done in six replicates, and fluorescence values were normalized by the OD600 of the corresponding cultures. (A) Fluorescence created by the addition of 10 μM Hoechst 33342 dye was recorded for 10 h. All values are normalized with respect to the mean FI values of the untreated wild-type E. coli cells (black line) recorded during the last 5 h when fluorescence remain unchanged. Fluorescence of wild type E. coli cells treated with the control-PPMO (gray dashed line) was similar to fluorescence of untreated wild type E. coli cells (black line). Fluorescence of acrA-PPMO-treated cells (2 μM acrA-PPMO: blue dashed line; and 10 μM acrA-PPMO: blue continuous line) and the acrA deletion cells (cyan line) reached slightly higher fluorescence levels at a higher initial rate. (B) Fluorescence measurements within the first 20 min (shown in A) demonstrate that the net influx rate of the Hoechst 33342 dye increases if acrA is deleted (cyan lines) or silenced (blue lines). This increase reflects the decreased efflux of the Hoechst 33342 dye by AcrAB-TolC complex. (C) Bar graph showing normalized mean fluorescence accumulation rates measured within first 5 min (B) after dye addition. Error bars represent standard deviations of six measurements. Accumulation rate increases by nearly 2.5 times (p < 0.001) when acrA is silenced or deleted. (D) Bar graph showing normalized mean final fluorescence values measured within the last 5 h (A) after dye addition. Error bars represent standard deviations of six measurements. Final fluorescence increases by nearly 1.3 times (p < 0.01) when acrA is silenced or deleted. (TIF) [file pbio.1002552.s006.tif]
